# Crystal structure of 3-[2-(4-methyl­phen­yl)ethyn­yl]-2*H*-chromen-2-one

**DOI:** 10.1107/S2056989014027790

**Published:** 2015-01-10

**Authors:** Ignez Caracelli, Stella H. Maganhi, Hélio A. Stefani, Karina Gueogjian, Edward R. T. Tiekink

**Affiliations:** a Departmento de Física, Universidade Federal de São Carlos, 13565-905 São Carlos, SP, Brazil; b Departamento de Farmácia, Faculdade de Ciências Farmacêuticas, Universidade de São Paulo, 05508-900 São Paulo-SP, Brazil; c Department of Chemistry, University of Malaya, 50603 Kuala Lumpur, Malaysia

**Keywords:** crystal structure, coumarins, asymmetric alkyne, hydrogen bonding, C—H⋯π inter­actions

## Abstract

The coumarin ring system in the title asymmetric alkyne, C_18_H_12_O_2_, is approximately planar (r.m.s. deviation of the 11 non-H atoms = 0.048 Å), and is inclined with respect to the methyl­benzene ring, forming a dihedral angle of 33.68 (4)°. In the crystal, supra­molecular zigzag chains along the *c*-axis direction are formed *via* weak C—H⋯O hydrogen bonds, and these are connected into double layers *via* weak C—H⋯π inter­actions; these stack along the *a* axis.

## Related literature   

For the biological activity of coumarins, see: Wu *et al.* (2009 ▸). For background to previous work on coumarins, see: Stefani *et al.* (2012 ▸). For a related structure, see: Elangovan *et al.* (2004 ▸). For synthetic details, see: Gueogjian (2011 ▸).
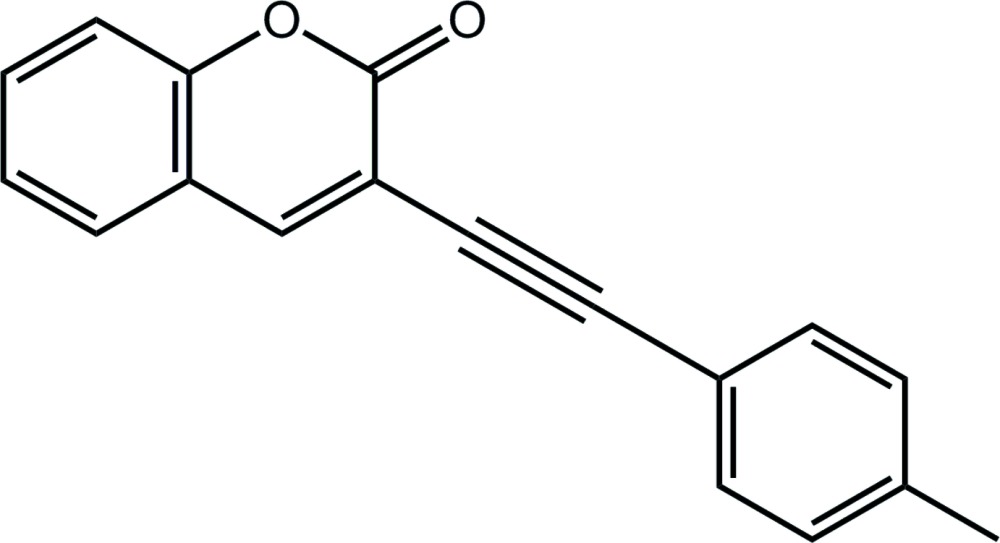


## Experimental   

### Crystal data  

C_18_H_12_O_2_
*M* *_r_* = 260.28
Monoclinic, 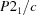

*a* = 8.4695 (2) Å
*b* = 10.6759 (2) Å
*c* = 14.5208 (2) Å
β = 98.093 (2)°
*V* = 1299.89 (4) Å^3^
*Z* = 4
Cu *K*α radiation
μ = 0.69 mm^−1^
*T* = 100 K
0.30 × 0.25 × 0.20 mm

### Data collection  

Agilent CCD diffratcometer diffractometer
Absorption correction: multi-scan (*CrysAlis PRO*; Agilent, 2011 ▸) *T*_min_ = 0.834, *T*_max_ = 1.000
5023 measured reflections
2664 independent reflections
2416 reflections with *I* > 2σ(*I*)
*R*_int_ = 0.015

### Refinement  

*R*[*F* ^2^ > 2σ(*F* ^2^)] = 0.038
*wR*(*F* ^2^) = 0.105
*S* = 1.04
2664 reflections
182 parameters
H-atom parameters constrained
Δρ_max_ = 0.25 e Å^−3^
Δρ_min_ = −0.21 e Å^−3^

### 

Data collection: *CrysAlis PRO* (Agilent, 2011 ▸); cell refinement: *CrysAlis PRO*; data reduction: *CrysAlis PRO*; program(s) used to solve structure: *SIR2014* (Burla *et al.*, 2015 ▸); program(s) used to refine structure: *SHELXL2014* (Sheldrick, 2008 ▸); molecular graphics: *ORTEP-3 for Windows* (Farrugia, 2012 ▸) and *DIAMOND* (Brandenburg, 2006 ▸); software used to prepare material for publication: *MarvinSketch* (ChemAxon, 2010 ▸) and *publCIF* (Westrip, 2010 ▸).

## Supplementary Material

Crystal structure: contains datablock(s) I, New_Global_Publ_Block. DOI: 10.1107/S2056989014027790/hg5424sup1.cif

Structure factors: contains datablock(s) I. DOI: 10.1107/S2056989014027790/hg5424Isup2.hkl

Supporting information file. DOI: 10.1107/S2056989014027790/hg5424Isup3.cml

**Fig. 1.**
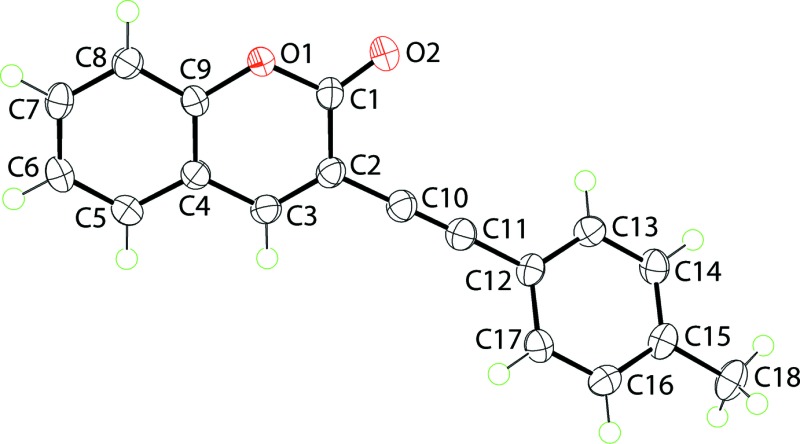
The molecular structure of the title compound showing the atom-labelling scheme and displacement ellipsoids at the 70% probability level.

**Fig. 2.**
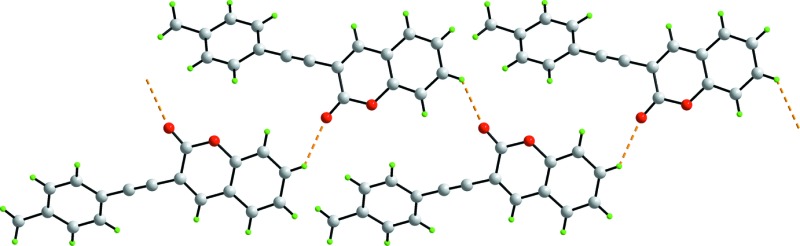
A view of the zigzag supramolecular sustained by weak C—H···O hydrogen bonds (orange dashed lines) and aligned along the c axis in the crystal packing.

**Fig. 3.**
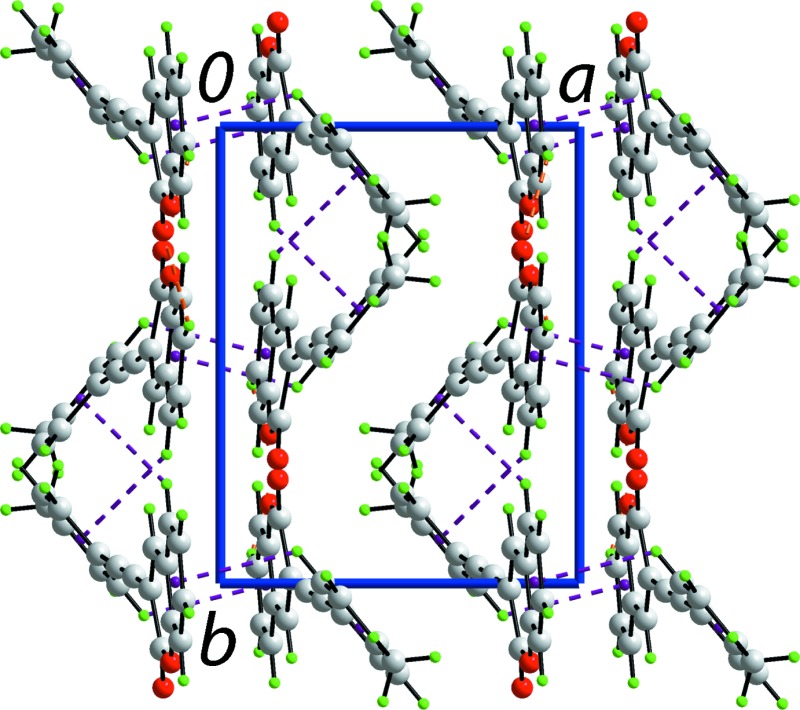
A view in projection down the c axis of the unit-cell contents. The weak C—H···O and C—H···π interactions are shown as orange and purple dashed lines, respectively.

. DOI: 10.1107/S2056989014027790/hg5424fig1.tif
The mol­ecular structure of the title compound showing the atom-labelling scheme and displacement ellipsoids at the 70% probability level.

c . DOI: 10.1107/S2056989014027790/hg5424fig2.tif
A view of the zigzag supra­molecular sustained by weak C—H⋯O hydrogen bonds (orange dashed lines) and aligned along the *c* axis in the crystal packing.

c . DOI: 10.1107/S2056989014027790/hg5424fig3.tif
A view in projection down the *c* axis of the unit-cell contents. The weak C—H⋯O and C—H⋯π inter­actions are shown as orange and purple dashed lines, respectively.

CCDC reference: 1040558

Additional supporting information:  crystallographic information; 3D view; checkCIF report

## Figures and Tables

**Table 1:** Hydrogen-bond geometry (, ) *Cg*1 and *Cg*2 are the centroids of the C4C9 and C12C17 rings, respectively.

*D*H*A*	*D*H	H*A*	*D* *A*	*D*H*A*
C7H7O2^i^	0.95	2.48	3.1425(14)	127
C13H13*Cg*1^ii^	0.95	2.94	3.4416(12)	115
C5H5*Cg*2^iii^	0.95	3.00	3.7780(13)	140

Symmetry codes: (i) 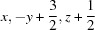; (ii) 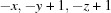; (iii) 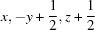.

## supplementary crystallographic information

### S1. Introduction

Coumarins are heterocycles presenting a wide range of different biological activities (Wu *et al.*, 2009). As part of our on-going inter­est in the synthesis of coumarin derivatives with biological activity (Stefani *et al.*, 2012) the title compound was synthesized.

### S2.1. Synthesis and crystallization

The title compound was prepared as per Gueogjian (2011). 3-Bromo coumarin (112.5 mg, 0.5 mmol), potassium tri­fluoro­borate salt (0.55 mmol), PdCl_2_(dppf).CH_2_Cl_2_ (41 mg, 10 mol%), *i*-Pr_2_NEt (0.3 mL, 1.5 mmol)and 1,4-dioxane/H_2_O (2/1, 3 mL), in aceto­nitrile (20 mL) were added to a two-necked round-bottomed flask equipped with a reflux condenser under N_2_. The reaction mixture was heated under reflux at 353 K, and was monitored by TLC and GC analysis. After the consumption of the 3-bromo­coumarin, the mixture was extracted twice with ethyl acetate (50 mL). The organic phase was separated, dried over MgSO_4_ and concentrated under vacuum. The residue was purified by flash chromatography (ethyl acetate/hexane 10:90). The title compound was obtained as a brown solid in 70% yield. Suitable crystals were obtained by slow evaporation from ethyl acetate/hexane.

### S2.2. Refinement

Carbon-bound H-atoms were placed in calculated positions (C—H = 0.95 to 0.98 Å) and were included in the refinement in the riding model approximation, with *U_iso_*(H) = 1.2–1.5*U_eq_*(C).

### S3. Results and discussion

The title compound, Fig. 1, is an asymmetric alkyne. The coumarin residue is approximately planar with the r.m.s. deviation of the 11 non-hydrogen atoms being 0.048 Å; the maximum deviations from their least-squares plane are 0.078 (1) and -0.066 (1) Å for the C2 and O2 atoms, respectively. Overall, the molecule is non-planar as seen in the dihedral between the fused ring system and the methyl­benzene ring of 33.68 (4)°.

The most closely related structure in the literature is of the derivative where the methyl group of the title compound has been replaced by an isoprop­oxy group (Elangovan *et al.*, 2004). In this case, with the exception of the terminal methyl groups, the molecule is planar with the dihedral angle between the 11 non-hydrogen atoms of the courmarin residue the benzene ring being 0.88 (6)°.

Weak coumarin-C_6_—C—H···O(exocyclic) hydrogen bonding gives rise to a supra­molecular chain aligned along the *c* axis, Table 1 and Fig. 2. The chains are connected into double layers, sustained by weak C—H···π inter­actions, that stack along the *a* axis with no specific inter­actions between them, Table 2 and Fig. 3.

### Crystal data

**Table:**

C_18_H_12_O_2_	*F*(000) = 544
*M**_r_* = 260.28	*D*_x_ = 1.330 Mg m^−^^3^
Monoclinic, *P*2_1_/*c*	Cu *K*α radiation, λ = 1.54184 Å
*a* = 8.4695 (2) Å	Cell parameters from 3107 reflections
*b* = 10.6759 (2) Å	θ = 3.1–76.1°
*c* = 14.5208 (2) Å	µ = 0.69 mm^−^^1^
β = 98.093 (2)°	*T* = 100 K
*V* = 1299.89 (4) Å^3^	Prism, dark orange
*Z* = 4	0.30 × 0.25 × 0.20 mm

### Data collection

**Table:**

Agilent CCD diffratcometer diffractometer	2416 reflections with *I* > 2σ(*I*)
Radiation source: SuperNova (Cu) X-ray Source	*R*_int_ = 0.015
ω scans	θ_max_ = 76.3°, θ_min_ = 5.2°
Absorption correction: multi-scan (*CrysAlis PRO*; Agilent, 2011)	*h* = −9→10
*T*_min_ = 0.834, *T*_max_ = 1.000	*k* = −6→13
5023 measured reflections	*l* = −17→18
2664 independent reflections	

### Refinement

**Table:**

Refinement on *F*^2^	Primary atom site location: structure-invariant direct methods
Least-squares matrix: full	Hydrogen site location: inferred from neighbouring sites
*R*[*F*^2^ > 2σ(*F*^2^)] = 0.038	H-atom parameters constrained
*wR*(*F*^2^) = 0.105	*w* = 1/[σ^2^(*F*_o_^2^) + (0.0605*P*)^2^ + 0.3358*P*] where *P* = (*F*_o_^2^ + 2*F*_c_^2^)/3
*S* = 1.04	(Δ/σ)_max_ < 0.001
2664 reflections	Δρ_max_ = 0.25 e Å^−^^3^
182 parameters	Δρ_min_ = −0.21 e Å^−^^3^
0 restraints	

### Special details

**Table:**

Geometry. All e.s.d.'s (except the e.s.d. in the dihedral angle between two l.s. planes) are estimated using the full covariance matrix. The cell e.s.d.'s are taken into account individually in the estimation of e.s.d.'s in distances, angles and torsion angles; correlations between e.s.d.'s in cell parameters are only used when they are defined by crystal symmetry. An approximate (isotropic) treatment of cell e.s.d.'s is used for estimating e.s.d.'s involving l.s. planes.

### Fractional atomic coordinates and isotropic or equivalent isotropic displacement parameters (Å^2^)

**Table:**

	*x*	*y*	*z*	*U*_iso_*/*U*_eq_	
O1	0.13803 (10)	0.67272 (7)	0.66818 (5)	0.0210 (2)	
O2	0.15964 (12)	0.72775 (8)	0.52430 (6)	0.0313 (2)	
C1	0.16397 (14)	0.64322 (11)	0.57962 (7)	0.0211 (2)	
C2	0.19557 (13)	0.51127 (11)	0.56003 (7)	0.0199 (2)	
C3	0.18389 (13)	0.42253 (10)	0.62585 (7)	0.0199 (2)	
H3	0.1980	0.3368	0.6115	0.024*	
C4	0.15055 (13)	0.45668 (10)	0.71666 (7)	0.0184 (2)	
C5	0.14038 (13)	0.36959 (11)	0.78810 (8)	0.0206 (2)	
H5	0.1504	0.2826	0.7763	0.025*	
C6	0.11585 (13)	0.40976 (11)	0.87544 (8)	0.0217 (2)	
H6	0.1078	0.3504	0.9233	0.026*	
C7	0.10299 (13)	0.53744 (11)	0.89331 (7)	0.0213 (2)	
H7	0.0880	0.5645	0.9538	0.026*	
C8	0.11176 (13)	0.62546 (11)	0.82380 (7)	0.0204 (2)	
H8	0.1033	0.7124	0.8360	0.025*	
C9	0.13313 (13)	0.58337 (10)	0.73620 (7)	0.0181 (2)	
C10	0.24056 (14)	0.48424 (11)	0.47093 (8)	0.0219 (2)	
C11	0.28627 (14)	0.46381 (11)	0.39787 (8)	0.0213 (2)	
C12	0.34093 (13)	0.43519 (11)	0.31108 (7)	0.0191 (2)	
C13	0.28464 (13)	0.50208 (11)	0.23036 (8)	0.0214 (2)	
H13	0.2132	0.5701	0.2333	0.026*	
C14	0.33248 (14)	0.46968 (12)	0.14599 (8)	0.0227 (3)	
H14	0.2941	0.5166	0.0919	0.027*	
C15	0.43582 (13)	0.36949 (11)	0.13914 (8)	0.0215 (2)	
C16	0.49343 (14)	0.30434 (11)	0.22028 (8)	0.0229 (2)	
H16	0.5653	0.2366	0.2172	0.027*	
C17	0.44814 (14)	0.33623 (11)	0.30511 (8)	0.0218 (2)	
H17	0.4897	0.2910	0.3595	0.026*	
C18	0.48231 (15)	0.32959 (13)	0.04694 (8)	0.0286 (3)	
H18A	0.4267	0.3821	−0.0027	0.043*	
H18B	0.4526	0.2417	0.0351	0.043*	
H18C	0.5978	0.3390	0.0486	0.043*	

### Atomic displacement parameters (Å^2^)

**Table:**

	*U*^11^	*U*^22^	*U*^33^	*U*^12^	*U*^13^	*U*^23^
O1	0.0288 (4)	0.0177 (4)	0.0170 (4)	0.0023 (3)	0.0054 (3)	0.0017 (3)
O2	0.0459 (6)	0.0265 (5)	0.0239 (4)	0.0070 (4)	0.0132 (4)	0.0074 (3)
C1	0.0237 (6)	0.0236 (6)	0.0164 (5)	0.0020 (4)	0.0046 (4)	0.0019 (4)
C2	0.0189 (5)	0.0235 (6)	0.0170 (5)	0.0023 (4)	0.0016 (4)	−0.0011 (4)
C3	0.0198 (5)	0.0201 (5)	0.0195 (5)	0.0020 (4)	0.0018 (4)	−0.0012 (4)
C4	0.0168 (5)	0.0201 (5)	0.0179 (5)	0.0009 (4)	0.0009 (4)	0.0001 (4)
C5	0.0202 (5)	0.0193 (5)	0.0217 (5)	0.0006 (4)	0.0008 (4)	0.0021 (4)
C6	0.0211 (5)	0.0252 (6)	0.0182 (5)	−0.0015 (4)	0.0003 (4)	0.0051 (4)
C7	0.0205 (5)	0.0282 (6)	0.0148 (5)	−0.0019 (4)	0.0007 (4)	−0.0009 (4)
C8	0.0216 (5)	0.0207 (5)	0.0187 (5)	−0.0006 (4)	0.0018 (4)	−0.0017 (4)
C9	0.0183 (5)	0.0194 (5)	0.0163 (5)	0.0001 (4)	0.0013 (4)	0.0023 (4)
C10	0.0223 (5)	0.0241 (5)	0.0189 (5)	0.0014 (4)	0.0018 (4)	−0.0002 (4)
C11	0.0213 (5)	0.0226 (5)	0.0197 (5)	−0.0006 (4)	0.0013 (4)	−0.0009 (4)
C12	0.0191 (5)	0.0214 (5)	0.0169 (5)	−0.0036 (4)	0.0027 (4)	−0.0027 (4)
C13	0.0196 (5)	0.0231 (6)	0.0209 (5)	0.0012 (4)	0.0013 (4)	−0.0014 (4)
C14	0.0218 (5)	0.0292 (6)	0.0164 (5)	−0.0008 (5)	−0.0006 (4)	0.0012 (4)
C15	0.0191 (5)	0.0278 (6)	0.0178 (5)	−0.0054 (4)	0.0030 (4)	−0.0048 (4)
C16	0.0211 (5)	0.0231 (6)	0.0250 (6)	0.0013 (4)	0.0053 (4)	−0.0022 (4)
C17	0.0229 (5)	0.0238 (6)	0.0184 (5)	−0.0002 (4)	0.0021 (4)	0.0023 (4)
C18	0.0274 (6)	0.0394 (7)	0.0200 (6)	−0.0036 (5)	0.0062 (5)	−0.0074 (5)

### Geometric parameters (Å, º)

**Table:**

O1—C1	1.3713 (13)	C8—H8	0.9500
O1—C9	1.3780 (13)	C10—C11	1.1995 (16)
O2—C1	1.2053 (14)	C11—C12	1.4353 (15)
C1—C2	1.4691 (16)	C12—C13	1.3978 (16)
C2—C3	1.3592 (15)	C12—C17	1.4036 (16)
C2—C10	1.4287 (15)	C13—C14	1.3873 (15)
C3—C4	1.4339 (14)	C13—H13	0.9500
C3—H3	0.9500	C14—C15	1.3945 (17)
C4—C9	1.3941 (15)	C14—H14	0.9500
C4—C5	1.4048 (15)	C15—C16	1.3958 (16)
C5—C6	1.3820 (15)	C15—C18	1.5090 (14)
C5—H5	0.9500	C16—C17	1.3833 (15)
C6—C7	1.3946 (17)	C16—H16	0.9500
C6—H6	0.9500	C17—H17	0.9500
C7—C8	1.3887 (16)	C18—H18A	0.9800
C7—H7	0.9500	C18—H18B	0.9800
C8—C9	1.3847 (15)	C18—H18C	0.9800
			
C1—O1—C9	122.56 (9)	C8—C9—C4	122.22 (10)
O2—C1—O1	117.33 (10)	C11—C10—C2	176.53 (12)
O2—C1—C2	125.32 (10)	C10—C11—C12	178.19 (12)
O1—C1—C2	117.35 (9)	C13—C12—C17	118.68 (10)
C3—C2—C10	123.44 (11)	C13—C12—C11	120.89 (10)
C3—C2—C1	119.92 (10)	C17—C12—C11	120.39 (10)
C10—C2—C1	116.62 (10)	C14—C13—C12	120.37 (11)
C2—C3—C4	120.84 (10)	C14—C13—H13	119.8
C2—C3—H3	119.6	C12—C13—H13	119.8
C4—C3—H3	119.6	C13—C14—C15	121.24 (10)
C9—C4—C5	118.20 (10)	C13—C14—H14	119.4
C9—C4—C3	118.30 (10)	C15—C14—H14	119.4
C5—C4—C3	123.46 (10)	C14—C15—C16	118.05 (10)
C6—C5—C4	120.34 (11)	C14—C15—C18	121.64 (11)
C6—C5—H5	119.8	C16—C15—C18	120.29 (11)
C4—C5—H5	119.8	C17—C16—C15	121.42 (11)
C5—C6—C7	119.99 (10)	C17—C16—H16	119.3
C5—C6—H6	120.0	C15—C16—H16	119.3
C7—C6—H6	120.0	C16—C17—C12	120.21 (10)
C8—C7—C6	120.84 (10)	C16—C17—H17	119.9
C8—C7—H7	119.6	C12—C17—H17	119.9
C6—C7—H7	119.6	C15—C18—H18A	109.5
C9—C8—C7	118.38 (10)	C15—C18—H18B	109.5
C9—C8—H8	120.8	H18A—C18—H18B	109.5
C7—C8—H8	120.8	C15—C18—H18C	109.5
O1—C9—C8	117.06 (10)	H18A—C18—H18C	109.5
O1—C9—C4	120.72 (9)	H18B—C18—H18C	109.5
			
C9—O1—C1—O2	177.09 (10)	C7—C8—C9—O1	178.68 (9)
C9—O1—C1—C2	−3.21 (15)	C7—C8—C9—C4	−1.88 (17)
O2—C1—C2—C3	−174.34 (12)	C5—C4—C9—O1	−178.37 (9)
O1—C1—C2—C3	5.98 (16)	C3—C4—C9—O1	3.95 (16)
O2—C1—C2—C10	6.73 (18)	C5—C4—C9—C8	2.21 (16)
O1—C1—C2—C10	−172.95 (10)	C3—C4—C9—C8	−175.47 (10)
C10—C2—C3—C4	175.00 (10)	C17—C12—C13—C14	0.91 (16)
C1—C2—C3—C4	−3.86 (17)	C11—C12—C13—C14	−176.91 (10)
C2—C3—C4—C9	−1.08 (16)	C12—C13—C14—C15	0.70 (17)
C2—C3—C4—C5	−178.62 (10)	C13—C14—C15—C16	−1.63 (17)
C9—C4—C5—C6	−0.84 (16)	C13—C14—C15—C18	177.00 (11)
C3—C4—C5—C6	176.70 (10)	C14—C15—C16—C17	0.99 (17)
C4—C5—C6—C7	−0.77 (17)	C18—C15—C16—C17	−177.67 (10)
C5—C6—C7—C8	1.12 (17)	C15—C16—C17—C12	0.60 (18)
C6—C7—C8—C9	0.18 (17)	C13—C12—C17—C16	−1.54 (17)
C1—O1—C9—C8	177.74 (10)	C11—C12—C17—C16	176.28 (10)
C1—O1—C9—C4	−1.71 (16)		

### Hydrogen-bond geometry (Å, º)

Cg1 and Cg2 are the centroids of the C4–C9 and C12–C17 rings, respectively.

**Table:**

*D*—H···*A*	*D*—H	H···*A*	*D*···*A*	*D*—H···*A*
C7—H7···O2^i^	0.95	2.48	3.1425 (14)	127
C13—H13···*Cg*1^ii^	0.95	2.94	3.4416 (12)	115
C5—H5···*Cg*2^iii^	0.95	3.00	3.7780 (13)	140

Symmetry codes: (i) *x*, −*y*+3/2, *z*+1/2; (ii) −*x*, −*y*+1, −*z*+1; (iii) *x*, −*y*+1/2, *z*+1/2.
