# Supplementary figures and images for: Crystal structure of 3-[2-(4-methyl­phen­yl)ethyn­yl]-2H-chromen-2-one
Source: Acta Crystallogr E Crystallogr Commun. 2015 Jan 10;71(Pt 2):o90–1. doi: 10.1107/S2056989014027790 (PMC4384545; doi:10.1107/S2056989014027790)

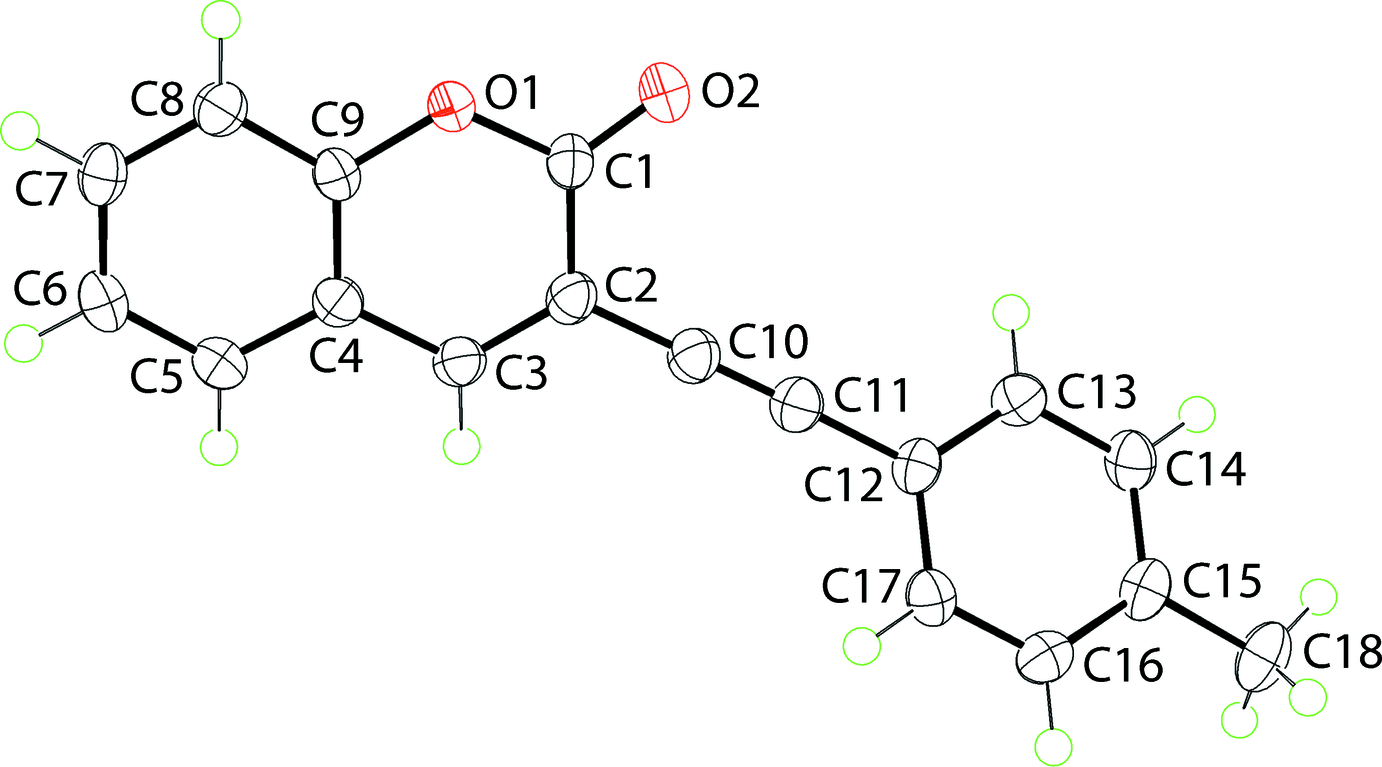

Supplement: Supplementary file 4 [file e-71-00o90-fig1.tif]

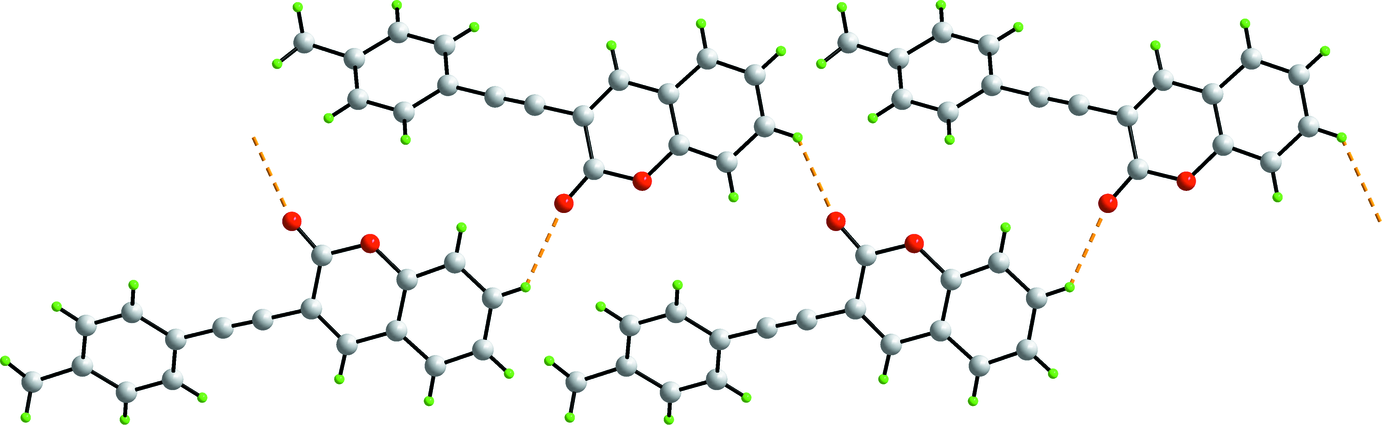

Supplement: Supplementary file 5 [file e-71-00o90-fig2.tif]

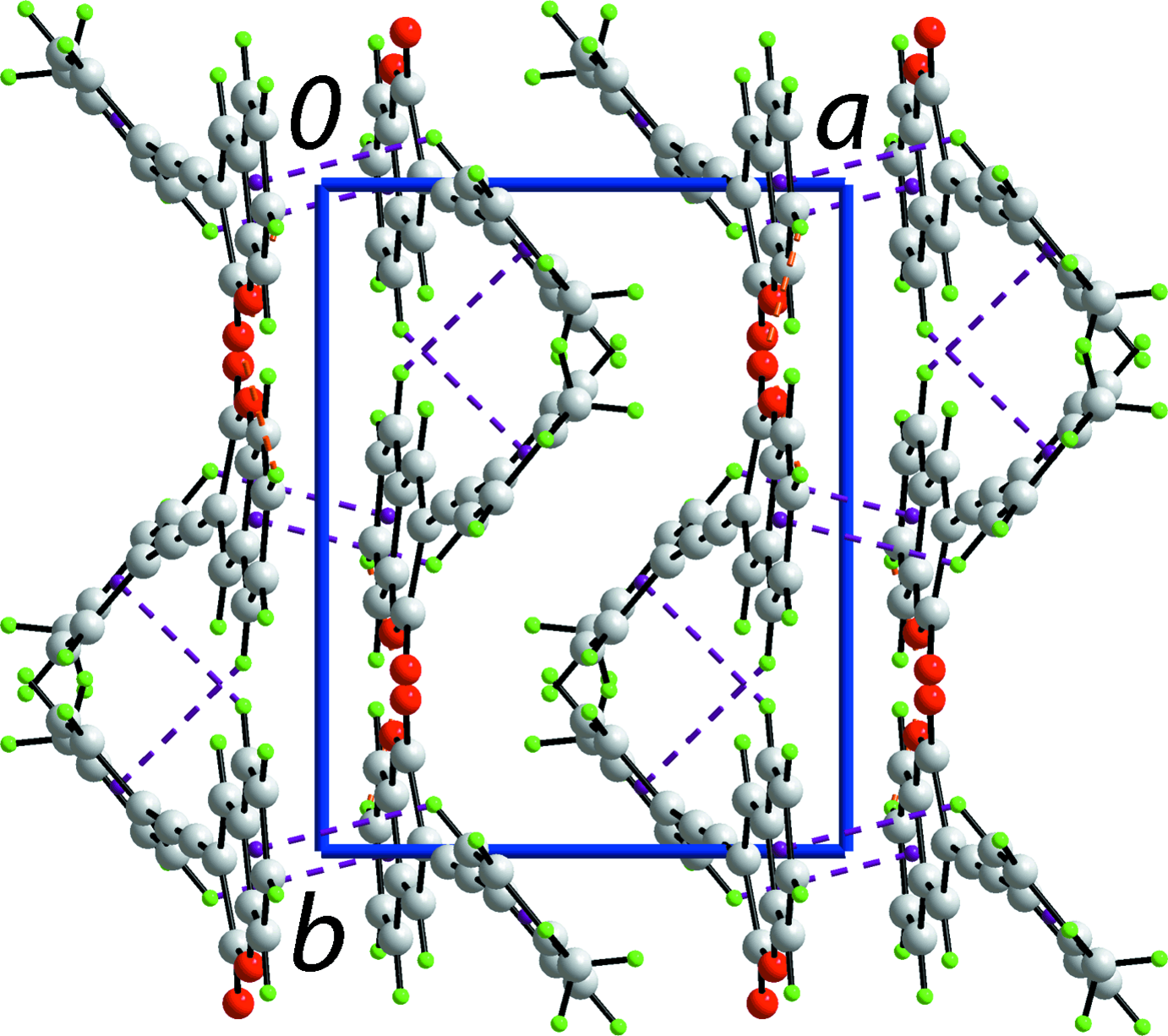

Supplement: Supplementary file 6 [file e-71-00o90-fig3.tif]
